# Application of postmortem imaging modalities in cases of sudden death due to cardiovascular diseases–current achievements and limitations from a pathology perspective: Endorsed by the Association for European Cardiovascular Pathology and by the International Society of Forensic Radiology and Imaging

**DOI:** 10.1007/s00428-022-03458-6

**Published:** 2022-12-24

**Authors:** Katarzyna Michaud, Christina Jacobsen, Cristina Basso, Jytte Banner, Britt M. Blokker, Hans H. de Boer, Fabrice Dedouit, Chris O’Donnell, Carla Giordano, Virginie Magnin, Silke Grabherr, S. Kim Suvarna, Krzysztof Wozniak, Sarah Parsons, Allard C. van der Wal

**Affiliations:** 1 University Center of Legal Medicine Lausanne - Geneva, Lausanne University Hospital and University of Lausanne, Lausanne, Switzerland; 2 Section of Forensic Pathology, Department of Forensic Medicine, University of Copenhagen, Copenhagen, Denmark; 3 Cardiovascular Pathology Unit, Department of Cardiac, Thoracic and Vascular Sciences and Public Health, University of Padua, Padua, Italy; 4 PAL Laboratory of Pathology, Dordrecht, The Netherlands; 5 Department of Forensic Medicine, Victorian Institute of Forensic Medicine, Monash University, Melbourne, Australia; 6 GRAVIT, Groupe de Recherche en Autopsie Virtuelle et Imagerie Thanatologique, Forensic Department, University Hospital, Rangueil, Toulouse, France; 7 Department of Radiological, Oncological and Pathological Sciences, Sapienza University of Rome, Rome, Italy; 8 Geneva University Hospital, University of Geneva, Geneva, Switzerland; 9 Department of Histopathology, Northern General Hospital, The University of Sheffield, Sheffield, UK; 10 Department of Forensic Medicine, Jagiellonian University Medical College, Krakow, Poland; 11 Department of Pathology, Amsterdam UMC, Academic Medical Center, Amsterdam, The Netherlands

**Keywords:** Sudden cardiac death, Aorta, Postmortem imaging, Autopsy, Coronary arteries, Myocardial infarction, PMMR, PMCTA

## Abstract

Postmortem imaging (PMI) is increasingly used in postmortem practice and is considered a potential alternative to a conventional autopsy, particularly in case of sudden cardiac deaths (SCD). In 2017, the Association for European Cardiovascular Pathology (AECVP) published guidelines on how to perform an autopsy in such cases, which is still considered the gold standard, but the diagnostic value of PMI herein was not analyzed in detail. At present, significant progress has been made in the PMI diagnosis of acute ischemic heart disease, the most important cause of SCD, while the introduction of postmortem CT angiography (PMCTA) has improved the visualization of several parameters of coronary artery pathology that can support a diagnosis of SCD. Postmortem magnetic resonance (PMMR) allows the detection of acute myocardial injury-related edema. However, PMI has limitations when compared to clinical imaging, which severely impacts the postmortem diagnosis of myocardial injuries (ischemic versus non-ischemic), the age-dating of coronary occlusion (acute versus old), other potentially SCD-related cardiac lesions (e.g., the distinctive morphologies of cardiomyopathies), aortic diseases underlying dissection or rupture, or pulmonary embolism. In these instances, PMI cannot replace a histopathological examination for a final diagnosis. Emerging minimally invasive techniques at PMI such as image-guided biopsies of the myocardium or the aorta, provide promising results that warrant further investigations. The rapid developments in the field of postmortem imaging imply that the diagnosis of sudden death due to cardiovascular diseases will soon require detailed knowledge of both postmortem radiology and of pathology.

## Introduction

Sudden cardiac death (SCD) is in many cases the first manifestation of heart disease and results from either a fatal arrhythmia or acute pump failure. Coronary artery disease (CAD) with evolving acute myocardial ischemia is the pathology most frequently observed in SCD in the adult population worldwide [1–3]. However, nearly all other cardiac diseases of significant severity, including non-ischemic diseases of the myocardium or conduction system, as well as valvar and aortic diseases, can be causes of SCD. In the absence of structural abnormalities, functional disorders such as primary arrhythmia due to ion channel disease should be suspected.

At present, the traditional autopsy is considered the “gold standard” for diagnosing natural causes of death, both in clinical and forensic pathology. However, postmortem imaging (PMI) has been used for diagnostic purposes in autopsy practice for a long time. Historically, X-rays were used in forensic practice for the detection of foreign bodies, such as firearm projectiles in burnt, putrefied, or mutilated corpses, or to collect evidence for the identification of unknown bodies. Postmortem angiography has long been applied to detect stenoses or obstructions of vessels, the origin of hemorrhages, and ruptured blood vessels/aneurysms, but this was initially limited to organs removed at autopsy [4]. Since the early 2000s, and especially in forensic practice, the panel of diagnostic postmortem imaging tools has expanded progressively with the introduction of whole-body imaging methods such as postmortem computer tomography (PMCT), postmortem CT angiography (PMCTA), postmortem magnetic resonance (PMMR), and postmortem magnetic resonance angiography (PMMRA). These can now also be applied in combination with image-guided biopsies [5–16]. Imaging techniques introduced new diagnostic perspectives, also in clinical pathology. Considering the cost of a conventional autopsy, the occupational health and safety hazards associated with it, and the objections from next of kin to performing an autopsy for religious or other reasons, PMI was recognized as a potential replacement for a conventional autopsy.

At present, the implementation of PMI in the diagnostic approach of a case varies according to the nation’s judicial system, and the type of autopsy (clinical versus forensic). But above all, its use relates to the questions it should answer. Depending on the degree of certainty sought to explain a sudden death (SD), the presumed cause of SD could be obtained from PMI combined with external examination and clinical data alone, without the necessity of an invasive autopsy. For example, it may suffice in a situation of clinically confirmed natural death at an older age when imaging detects a hemopericardium. This approach allows some countries to triage cases for a non-invasive examination only. In other instances, a full invasive autopsy is required, for example, when precise histological diagnosis or dating of a pathological lesion is needed (which includes also cases involving medical responsibility) or when a genetic cause is suspected in a setting of sudden unexplained arrhythmic death (SAD). These cases require a protocolized investigation of the heart at autopsy and the collection of materials for histology, toxicology, and genetics [2].

In summary, three different scenarios can nowadays apply to a postmortem investigation: a full autopsy, a PMI only, and a combined approach. Different pathological entities will display different diagnostic yields with either autopsy or various imaging techniques. However, up to now, the diagnostic yield of autopsy and PMI have been published only fragmentarily throughout the recent literature (be it pathology or radiology), and large randomized trials do not exist. Moreover, some studies refer to diagnostic achievements of the novel in vivo imaging techniques, which are obviously not realistic in a postmortem setting [17, 18].

This article reviews the current diagnostic possibilities of PMI for cardiovascular lesions that can be considered important causes of SCD. As a point of departure, all cardiac diseases that can potentially be involved in SCD will be evaluated. Such a listing has been published by AECVP, which also categorizes diseases according to their degree of certainty in explaining the death of a victim (i.e., be it certain, probable, or uncertain) [2, 19]. This approach may provide insights into what extent, in which situations (clinical or forensic investigation), and for which diseases the various postmortem imaging techniques are now useful to explain sudden cardiac death.

### PMI: currently most applied methods

Currently, most applied PMI techniques are traditional plain X-rays, PMCT, and PMMR, of which the latter two can be used in combination with angiography (PMCTA and PMMRA, respectively). Naturally, PMI is not the same as in vivo imaging of tissues or organs. In a postmortem setting, functional parameters or uptake of molecular markers cannot be applied, and only the morphological and volumetric aspects can be investigated. It should be emphasized that training in radiology and postmortem imaging by PMCT/PMCTA and PMMR is necessary to interpret the findings correctly. Also, similar to an autopsy, it is crucial to take the clinical history, circumstances of death, family history, and drug history into account when interpreting the PMI findings. The most relevant aspects of the various radiological methods are summarized in Table 1 (adapted from Michaud et al. [20]) considering the literature [6, 9, 21–34].

**Table 1:** Summary of radiological methods used in postmortem heart examination

Method	Advantages	Disadvantages	References
Plain X-rays	Fast examination Simple data storage Relatively low maintenance costs Easy visualization of the skeletal system Detection of radiopaque foreign bodies (e.g., medical devices) Visualization of the heart size, and large calcifications of the aorta	Exposure to ionizing radiation No 3D reconstructions Very limited visualization of soft tissue, such as blood vessels, heart valves, and myocardium Superimposed image Quality strongly depends on the acquisition	[21]
PMCT	Fast examination Allows for 3D reconstructions Relatively low maintenance costs Excellent visualization of skeletal system and gas collections Visualization of the heart size, calcifications, hemopericardium, and radiopaque devices Detection of radiologically suspected areas for postmortem histological examination (guided biopsies)	Exposure to ionizing radiation Data storage Limited visualization of cutaneous tissue, organs, and vessels	[22–26]
PMCTA	Visualization of vessels, possible evaluation of stenosis and occlusions Relatively fast examination Allows for 3D reconstructions of vessels Guided biopsies possible	Exposure to ionizing radiation Maintenance costs Data storage Histological and radiological artifacts Technically more challenging	[9, 21, 27, 28]
PMMR	Excellent visualization of organs and other soft tissues Allows for 3D reconstruction No radiation	Time consuming High maintenance costs Need specific building construction 3D reconstructions need isotropic sequences Data storage Limited interpretation of the lumen of the coronary arteries Interpretation is limited by postmortem artifacts, resuscitation, body temperature Specific care if internal metallic objects are present (stents, devices) Guided biopsies are technically complex	[6, 29–31]
PMMRA	Visualization of organs and other soft tissue, blood vessels, and vascular lumen No radiation Allows for 3D reconstructions of vessels	Time consuming High maintenance costs Need specific architectural construction 3D reconstructions need isotropic sequences Data storage Interpretation is limited by postmortem artifacts, resuscitation, body temperature Guided biopsies are technically complex Metallic instruments cannot be used	[21, 32–34]

Adapted from Michaud et al. [20]. *CTR*, cardiothoracic ratio; *PMCT*, postmortem computed tomography; *PMCTA*, postmortem computed tomographic angiography; *PMMR*, postmortem magnetic resonance; *PMMRA*, postmortem magnetic resonance angiography; *3D*, three-dimensional

## Postmortem imaging of cardiovascular structures potentially involved in SCD—state of the art

### Coronary arteries

Acute total or subtotal thrombotic or hemorrhagic occlusion of one of the major epicardial coronary arteries induces an acute ischemic change in the downstream myocardium. Fatal ventricular arrhythmias leading to sudden collapse can develop very early after the onset of an ischemic injury. Therefore, the finding of acute thrombotic occlusion at autopsy, nearly always superimposed on a disrupted atherosclerotic plaque, is considered a certain cause of death.

However, coronary artery disease (CAD) is widespread among adults, and many individuals over the age of 30 years have at least some atherosclerotic plaque formation in their coronary arteries. Recognizing the acutely thrombosed (red) types of occlusion, which are considered to represent a certain cause of acute coronary death, is therefore crucial. The layered aspect of an antemortem thrombus and its attachment to an eroded or ruptured atherosclerotic plaque can be used to histologically differentiate between acute (fresh) thrombus and postmortem clot. Acute thrombotic occlusions should also be distinguished from chronic total occlusions (CTO): fibrocellular calcified occluding lumen masses associated with organized thrombosis of at least months old, which are not uncommonly found at autopsy [2, 35, 36].

Moreover, albeit more rarely, other coronary diseases such as vasculitis or dissection can be complicated by acute thrombotic occlusion with the inherent risk of ischemia-related ventricular fibrillation (VF). Histologically, these can only be distinguished from atherosclerotic obstruction. Table 2 lists the various coronary artery diseases that can be diagnosed at autopsy (including histological analysis) and their categorization according to their degree of certainty to explain the onset of SCD.

**Table 2:** Coronary artery pathologies and their degree of certainty to explain the onset of SCD: (adapted from Basso et al. [2])

Certain	Probable	Uncertain
Acute total or subtotal thrombotic occlusion Mechanic obstruction of coronary ostium Anomalous origin of coronary from the pulmonary trunk	Significant (> 75%) stenosis, in combination with other factors leading to oxygen demand–supply mismatch (exercise, cardiac dilation, etc.) Aberrant origin of LCA with inter-arterial course	Minor congenital abnormalities of coronary arteries (including myocardial bridging) Small vessel disease

In PMI, it is impossible to detect a coronary occlusion without visualization of the vascular lumen. Only calcifications, which are the radiological features considered most closely related to atherosclerosis, can be observed in PMCT. Clinically, the extent of coronary calcification can be evaluated by means of coronary artery calcium scoring (CACS), commonly referred to as the Agatston method [37]. Postmortem CACS can be assessed by direct calcium quantification [38]. In a recent Australian study, it was reported that about one-third of patients who died from acute CAD had zero CACS in postmortem imaging [39]. This paradoxical discrepancy between imaging and autopsy findings can be explained by considering the histological aspect of fatal coronary plaques in such instances, which are usually highly stenosing eroded lesions composed of fibrocellular but not calcified tissue adjacent to the luminal thrombus. These lesions occur more often at a younger age [40, 41]. These results suggest that CACS graded “zero or low” cannot rule out the presence of extensive stenosis and that CACS can neither confirm nor exclude death due to acute coronary artery disease (ACAD). This is in line with the study by Wagensveld et al. reporting that the total Agatston calcium score has a good diagnostic value for chronic myocardial infarction (MI) (area under the curve (AUC), 0.74; 95% confidence interval (CI), 0.64–0.84), but not for acute MI (AUC, 0.60; 95% CI, 0.48–0.72) [42].

Various PMI modalities have been studied in an effort to visualize the vessel’s lumen [21, 27, 33, 43–47].

**Fig. 1:**
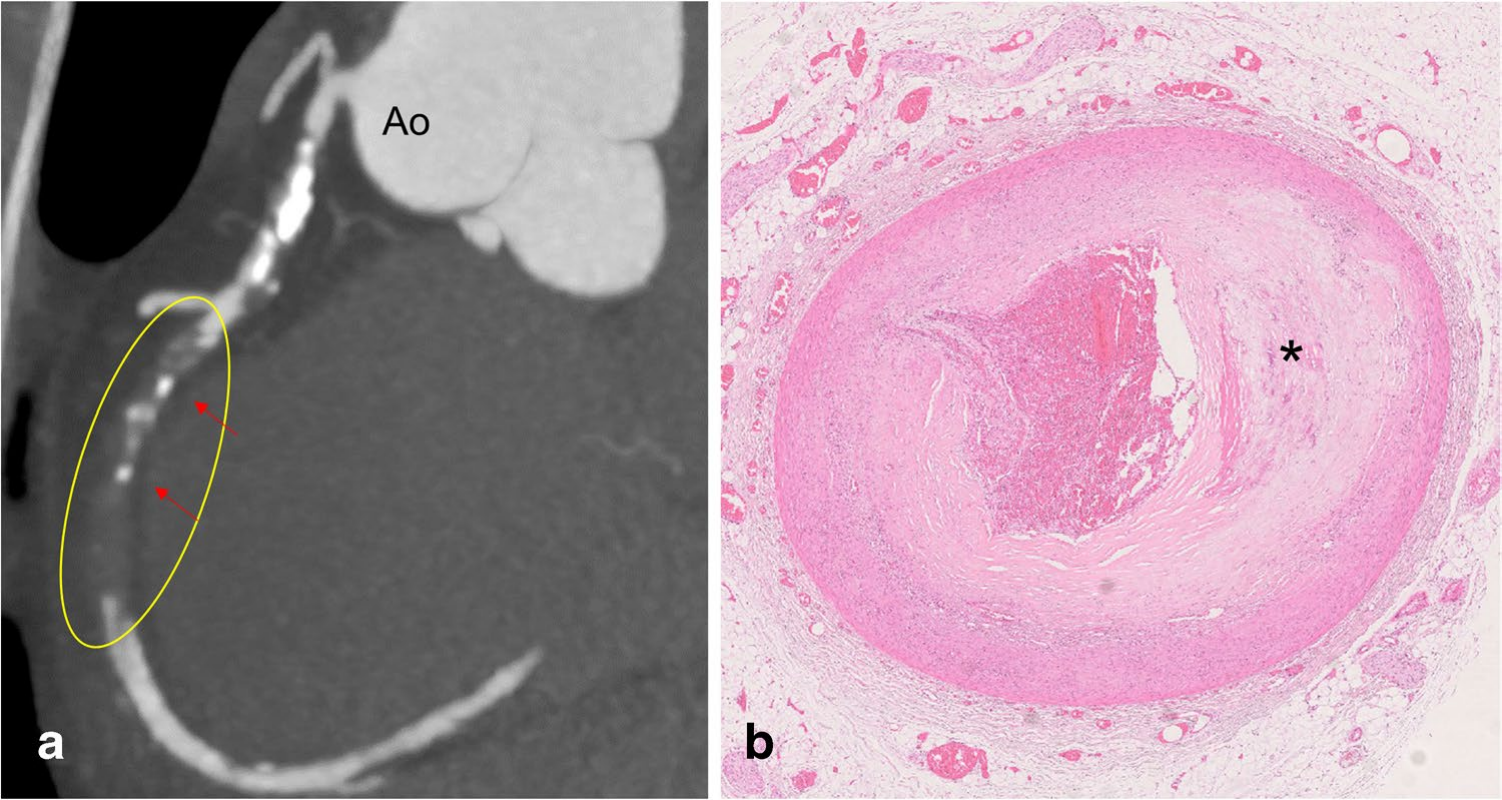
SCD in a 42-year-old male due to obstructive CAD, **a** PMCTA image of the heart and aortic root (Ao), with vascular reconstruction of the right coronary artery showing a large opacification defect (yellow ellipse) interpreted as occlusive atherosclerotic disease, with the presence of spotty calcifications (red arrows). **b** Histology (HE stain) of a cross-section taken from the proximal part of the occlusion shows atherosclerotic plaque with surface disruption and adjacent thrombotic occlusion of the vessel lumen. The asterisk indicates microcalcifications inside the plaque

**Fig. 2:**
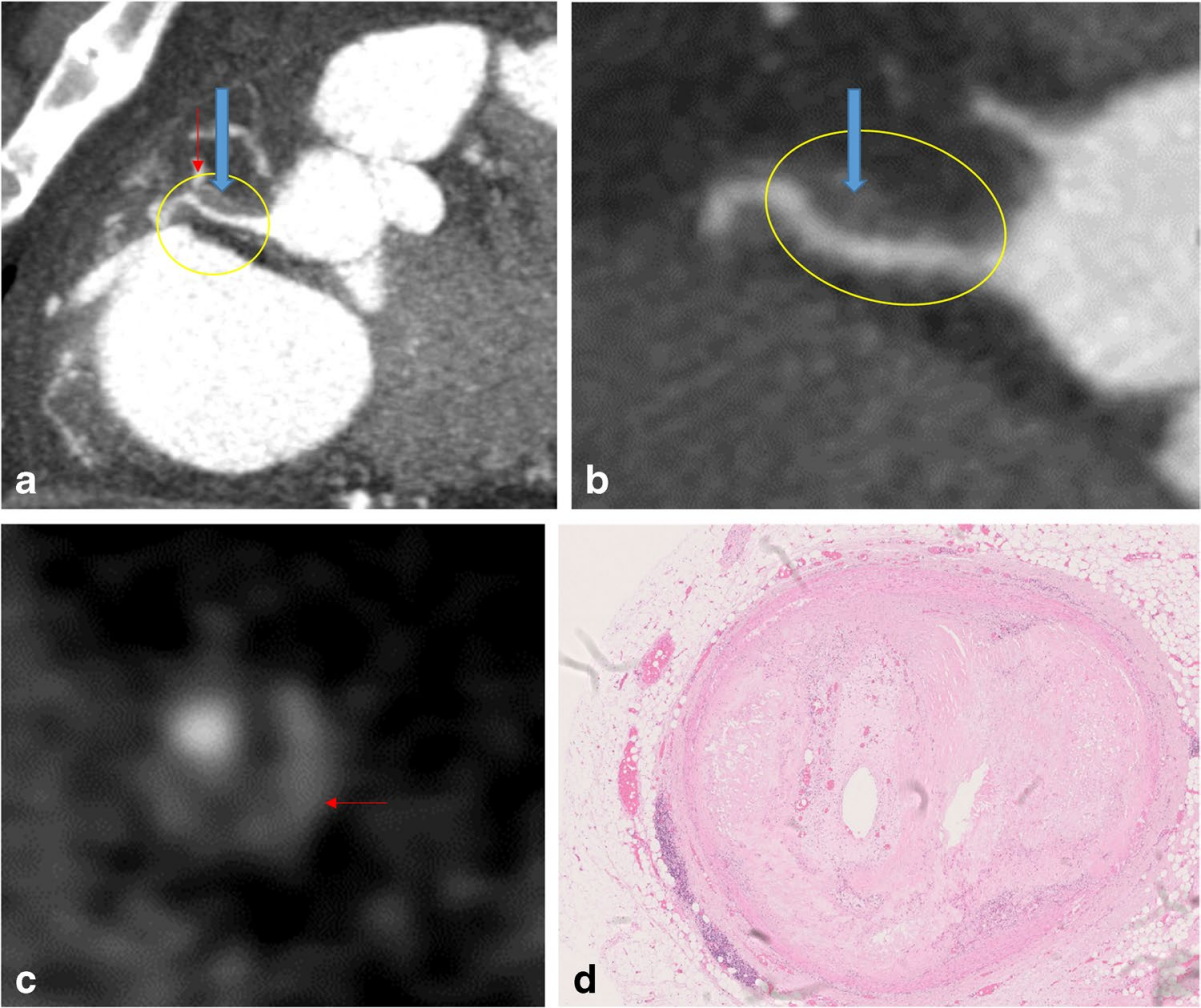
SCD in a 52-year-old male with severe CAD. **a**–**c** PMCTA images showing a coronal-oblique plane (**a**) and linear vascular reconstruction (**b**) of the right coronary artery with atherosclerotic plaque (yellow ellipse) and positive remodeling (blue arrows). **c** Detail of the axial vascular plane with the napkin-ring sign of plaque (red arrows). **d** Histology (HE stain) of a cross-section through same coronary artery segment showing subtotal chronic occlusion due to fibrolipid plaque tissue with microvascularization; no acute thrombus was noticed

The best results for identifying coronary stenosis or occlusion have been obtained with the use of PMCTA. In clinical radiology, the concept of plaque vulnerability has been established [48–51], referring to radiological signs of coronary plaques with a higher risk to develop the acute coronary syndrome. These are napkin-ring signs, positive remodeling, spotty calcification, and low plaque attenuation. Such signs can be detected by PMI and especially PMCTA, but the described cases in the literature are rare [47, 52]. Also, several shortcomings and pitfalls need to be taken into account. The differentiation between a vital and postmortem occlusion and a chronic total occlusion (CTO) is difficult if not impossible with postmortem imaging. Only the presence of local collateral circulation (bridging around the occlusion or recanalization inside the lumen) could provide evidence of chronicity [27]. Also, due to partial preservation of perfusion, mural non-occlusive thrombosis (actually representing the cause of a SCD) can be missed on PMI [27, 47]. One should be aware that older lytic or organized parts of a thrombus (which may be of medico-legal significance) and identification of acute plaque hemorrhages, dissection, or recanalization in areas of critical stenosis or occlusion is unreliable (if not impossible) with PMCTA (see Figs. 1 and 2), all of which need histological verification [53–56].

Only a few authors have reported on the PMMR-based diagnosis of coronary artery disease-related deaths [57, 58]. The occurrence of chemical shift artifacts (an *MR artifact that occurs at the interface between fat and water*) along the coronary arteries on non-contrast PMMR was proposed to evaluate eventual acute coronary artery disease in SCD cases [58]. A specific signal alteration within the lumen of the vessel that differed from the normal postmortem blood appearance was interpreted as an image of coronary thrombosis [57]. PMMRA has been investigated in experimental studies, combining the visualization of the coronary vasculature and MRI-derived myocardial tissue characteristics in order to visualize potential coronary occlusions related to myocardial infarction (i.e., edema). Results are promising, but further research is needed in order to validate the technique in a diagnostic postmortem setting [32, 46, 59].

According to the AECVP guidelines on SCD, the postmortem cut-off to consider coronary stenosis as severe/significant is 75% [2]. It should be noted, however, that such stenotic plaques can only be considered significant to explain death when other factors compromising the oxygen demand and supply of the myocardium are also present around the time of death. Such factors can be physiological (exercise) or pathological (such as cardiac dilatation, hypovolemia, and others). Histological examination of the plaque (to evaluate the presence or absence of thrombus) should be performed at autopsy. In the absence of a thrombus, a search for concomitant factors evoking acute coronary ischemia is crucial for diagnosis, both in the case of autopsy and in the case of evaluating postmortem images. Of course, other potential causes of death have to be excluded.

In postmortem practice, histological evaluation tends to overestimate the degree of stenosis by 25–30% when compared with clinical angiographic methods [60]. Therefore, PMCTA could give a better impression of the stenosis due to mimicking arterial pressure. Morgan et al. [56], reported that there was an agreement between PMCTA and histological examination of the culprit lesion at autopsy, representing a critical stenosis of > 75%, with a sensitivity of 85.7% and specificity of 91.5%. Discrepancies between autopsy and PMCTA came up in this study when histology was assessed on a segmental basis; in regions of densely calcified vessels assessed pathologically as critical stenosis, PMCTA showed markedly increased patency. Such areas likely relate to positive remodeling of the vessel wall at sites of plaque formation. Singh et al. investigated the sensitivity and specificity of PMCTA versus histopathology at autopsy in diagnosing coronary artery stenosis over 70% using water-based contrast media, which revealed a sensitivity of 61.5% and specificity of 91.7%. In addition, the authors commented that pericardial hematoma and stented coronary arteries limit the diagnostic value of PMCTA. In the studies mentioned above, thrombotic lesions could not be identified [56, 61].

It is also important to emphasize a relatively rare stenotic coronary lesion, occurring only in the young (< 40 years), and frequently observed in forensic settings. These lesions are hypercellular with no calcifications, with local negative remodeling and a small lumen area, but usually lack thrombosis at the time of death/autopsy. It is proposed that vascular smooth muscle cell hyper-reactivity is a pathophysiological substrate for spasms leading to death in these individuals [62, 63]. These lesions should be visible at PMCTA, as high-grade stenosis without calcification.

Acute coronary ostial occlusions and the anomalous origin of one coronary artery from the pulmonary trunk, which are both considered certain causes of SCD, can be detected fairly easily by angiographic methods. Anomalous LCA origin from the right sinus with an interarterial course, which is considered a probable cause of SCD, can also be detected with angiographic methods. Most minor abnormalities of the coronary artery tree, as listed in Table 2, can be detected with the use of various angiographic methods. These minor abnormalities include aberrant ostia and the course of epicardial arteries, such as myocardial bridging. Such findings cannot reliably explain the onset of SCD in a patient and should therefore not be overinterpreted as the cause of death. Antemortem occurrence of coronary spasms as a cause of death is difficult if not impossible to detect at both autopsy and postmortem imaging.

### Myocardium

Significant myocardial pathology is a major cause of SD and is, in most cases, related to myocardial ischemia/infarction. Ischemic necrosis should be discriminated from other forms of myocardial injuries, such as resuscitation ischemia/reperfusion injury, myocarditis, cardiomyopathies, or endogenous catecholamine-related myocyte death, which is possible with the use of enzymatic and/or (immune) histological methods at autopsy [36, 64]. However, very early changes, such as edema, are difficult to interpret reliably. Furthermore, for a proper diagnosis of most inherited or acquired cardiomyopathies and cardiac tumors, histopathological examination is indispensable [64]. In every case, it is important to interpret the findings in the context of the clinical history. Table 3 lists the various myocardial causes of SCD that can be diagnosed at autopsy (including subsequent histology) in most cases.

**Table 3:** Myocardial causes of SCD, including protruding cavitary tumors and the conduction system categorized according to their degree of certainty to explain the onset of SCD (adapted from Basso et al. [2])

Certain	Probable	Uncertain
Acute myocardial infarction Acute diffuse myocarditis (any morphological type)	Chronic ischemic heart disease (ischemic scar, any cause) Cardiomyopathies (genetic and acquired) Multifocal myocarditis Sarcoidosis and storage diseases Intramural ventricular/septal tumors (of the working myocardium and conduction tissues) Large intracavitary tumors AV anomalous pathways with previously recorded ECG tracing	Focal myocarditis Idiopathic LV hypertrophy Hypertensive heart disease Hypertrabeculation (non-compacted) myocardium Atrial septum lipoma (intramural) Fibrosis of RBB and LBB (Lenègre disease)

*AV*, atrioventricular; *LBB*, left bundle branch; *LCA*, left coronary artery; *LV*, left ventricle; *RBB*, right bundle branch; *SCD*, sudden cardiac death

### General considerations: clinical vs. postmortem MRI imaging of the myocardium

The myocardium, as a soft tissue, can be best visualized radiologically by using MRI, both during life and the postmortem setting [20, 29, 65].

In a clinical setting, MRI is considered useful for the detection of structural abnormalities in the myocardium as well as in situations when there is a need for high-resolution structural imaging, e.g., valvular or adult congenital heart disease [66]. The use of contrast agents, typically gadolinium, with T2- and T1-weighted sequences [67] or quantitative mapping of T1 and T2 scans, offers the possibility of evaluating myocardial tissue change [68–71]. T1 and T2 mappings are based on parametric quantitative sequences that provide tissue-specific T1 and T2 values that can be used to evaluate tissue composition [68, 69]. These sequences provide the possibility to quantify diffuse and focal myocardial disease processes (such as edema, myocyte necrosis, scars, and storage-related products) [72, 73]. As such, they are useful for diagnosing ischemic heart disease, myocarditis, storage diseases, and cardiomyopathy-related pathological changes based on tissue-specific reference values of normal tissue [68–70].

Patterns of enhancement and cine imaging applied in vivo are currently also under investigation in PMI. However, limitations exist. For instance, gadolinium cannot be used to enhance pathologic areas in the myocardium. Also, applying T1 and T2 mapping sequences poses problems, as it is necessary to establish a certain amount of specific normal reference values for the local scanner and magnet to be able to evaluate relaxation times correctly [68, 71, 74–76]. In a postmortem setting, increased water content (edema) of myocardial tissue, as seen in acute myocardial injuries, causes prolongation of relaxation times of both T1- and T2-weighted sequences. Even if both T1- and T2-weighted sequences have proven useful in a postmortem setting, the signal behavior and the reading of the alterations are partially reader dependent. For all PMMR studies, postmortem artifacts as well as different temperature ranges of bodies can pose difficulties for image contrast on T1- and T2-weighted images as well as for establishing reference values in quantitative studies [74–80].

### Acute myocardial infarction

**Fig. 3:**
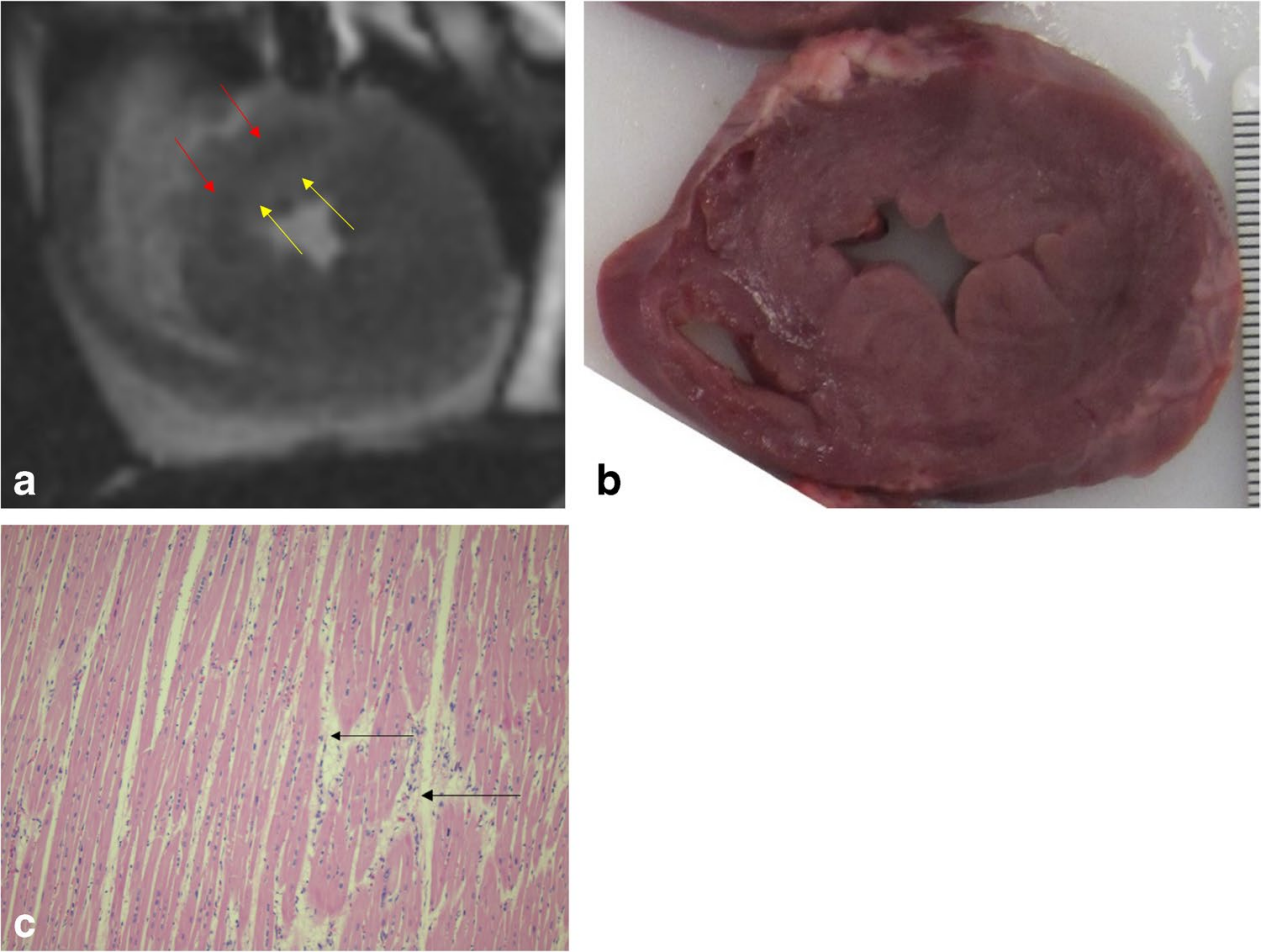
Animal model of myocardial infarction, evoked by occlusion of the left anterior descending artery of < 60 min. **a** PMMR—short axis plane—with a hypointense region (red arrows) in the septum and frontal wall of the left ventricle surrounded by a hyperintense region (yellow arrows). **b** Midventricular transversal slice of the fresh heart with normal gross structure of myocardium. **c** Histology (HE stains) of left ventricle anterior wall showing interstitial edema between myocardial fibers (arrows)

As mentioned above, PMMR can be valuable in diagnosing myocardial edema as a sign of ischemia in the course of myocardial infarction [57, 74, 80–82]. It should be underlined however that edema can be a sign of any type of myocardial injury, not only of myocardial infarction [36, 64]. At the onset of injury (including ischemic), the first cell changes and edema develops. The accumulation of intercellular water content increases signal intensity on T2-weighted PMMR images [83]. The first studies on the subject were on small populations or case reports, and the temperature-dependent variation of signal intensity on T1-weighted, proton density images, and to a lesser extent on T2-weighted images, became obvious as a limitation [57, 82, 84]. The first large prospective study using a 3 T MR scanner, conducted by Jackowski et al. [80], confirmed that PMMR could diagnose acute, subacute, and chronic infarction, correlated to the myocardial findings on autopsy. Only cases with myocardial lesions on PMMR were included in the study. The authors found that the ischemic area in the acute phase was characterized by a hypointense center with a hyperintense margin on T2-weighted images. A subacute infarction area produced an overall hyperintense region, while chronic infarction with fibrotic areas resulted in a uniform area of signal loss on T2-weighted images. Several cases with the so-called hyperacute lesions on T2-weighted images were found [80, 84], interpreted as ischemic lesions with an age of under 1 h. Most of these lesions could not be confirmed using histopathology, but several cases corresponded with macroscopic findings of coronary thrombus at autopsy, confirming a highly likely ischemic event. Animal model studies have shown that acute ischemic events with > 4 h of coronary artery ligation manifest as hyperintense areas in T2-weighted images [85] (see also Fig. 3). In another study, 60 min of coronary artery occlusion and 120 min of reperfusion resulted in a large central zone of intermediate hyperintensity and a zone of increased intensity in the periphery of the ischemic tissue on T2-weighted images [81]. Zech et al. have used a quantified MRI synthetic approach on a 3-T MR scanner to analyze T1, T2, and proton density relaxation times, thereby providing a reader-independent approach to evaluating the age of myocardial infarction. Despite the low number of cases, these results indicated that it is possible to differentiate quantitatively between histologically verified early acute (> 6 h) infarctions and older stages of myocardial infarction. The authors further commented that the evaluation of ischemic myocardial lesions using signal behavior in T1- or T2-weighted images was found to be partially reader-dependent [74, 75]. The same approach was used on a 1.5-T MR scanner with similar results [76]. However, it was concluded that when postmortem quantitative cardiac MRI will be used in the future for routine diagnosis of myocardial infarction, it is mandatory that the quantitative values of the different histopathological age stages of myocardial infarction need to be known for 1.5- and 3-T applications. Moreover, it remains unclear whether quantitative cardiac MRI is feasible for the detection of myocardial infarction aged less than 6 h without visible pathologic alterations in conventional histology [74].

### Myocarditis

Clinically acute myocarditis is diagnosed using T2-weighted sequences, sometimes supplemented by the use of gadolinium and sequences for T1 mapping [72, 86], depending on the age of the inflammatory changes. No postmortem studies have been published on the evaluation of myocardial inflammatory diseases. As a consequence, it is not yet established whether acute diffuse, multifocal, or focal myocarditis can be diagnosed on PMMR. As exemplified by quantitative PMMR studies on myocardial infarction [74–76], it seems plausible that T2 and T1 mapping sequences might be of use in a postmortem setting to detect myocarditis-related edema, but this needs to be established in future studies.

### Myocardial injuries and minimally invasive autopsy

Recently, several studies have evaluated the use of minimally invasive autopsy (MIA) in cardiac death victims and specifically focused on the detection of myocardial injuries, including myocardial ischemia. The term MIA refers to an autopsy in which the classical opening of body cavities is replaced by integrating various postmortem imaging techniques, including tissue biopsy and/or angiography.

In 2009, Weustink et al. showed that MIA by PMCT and PMMR failed to demonstrate ischemic heart disease [87]. Biopsies in this pilot study were either ultrasound guided or, including those obtained from the heart, unguided. A review by Blokker et al. in 2016 compared non-invasive postmortem examination, or MIA, to the conventional autopsy of suspected adult natural deaths. They reported that the combination of PMCT and PMMR was the best non-invasive method, but that minimally invasive autopsy methods surpassed the diagnostic accuracy of the non-invasive methods. The highest sensitivity for the cause of death (90.9%, 95% CI: 74.5; 97.6) was achieved by combining PMCT, PMCTA, and biopsies [5]. In a prospective validation study, Blokker et al. then compared an updated MIA protocol, utilizing improved imaging modalities, PMCT-guided biopsies, and stereotactic brain biopsies, to conventional autopsy [16]. They proved to have equal performance in establishing causes of death. Within this cohort, Wagensveld et al. focused on the diagnostic accuracy for ischemic heart disease (IHD) [42]and found that the combination of PMMR and extensive sampling by targeted biopsies from different regions of the myocardium had the highest accuracy. They achieved a sensitivity and specificity of 0.90 and 0.75 for diagnosing chronic myocardial infarction and 0.97 and 0.95 for diagnosing acute myocardial infarction. In another study, Wagensveld et al. also stated that MIA can answer clinical questions and detect major diagnoses properly. The diagnostic yield and clinical utility for PMCT and PMMR as stand-alone modalities were however low [88].

### Cardiac size and hypertrophy

In pathology, heart weight and dimensions are important indicators of cardiac disease and the potential onset of sudden death [89]. An accurate diagnosis of hypertrophy using postmortem imaging would be an important indicator to decide whether morphological alterations such as hypertrophy and/or dilatation are present. Clinically, the cardiothoracic ratio (CTR) is used to establish cardiomegaly using a threshold ratio of 0.5 to define cardiomegaly. This ratio is obtained under specific technical conditions that cannot be achieved postmortem. Several studies have shown that the attempt of using the same threshold ratio on PMI, especially PMCT, is not sufficiently accurate [90], particularly since CTR is influenced by body mass index (BMI), dilatation of the atria and ventricles (especially the right atrium [91]), and gas accumulation. It has been shown that dilatation of the heart varies depending on the mechanism of death in that the cardiac dilatation index (CDI) is greater in, e.g., fire-related deaths, hypothermia, and fatal intoxications and lower in cases of mechanical asphyxiation and drowning [92]. It may also vary with postmortem interval, inducing tissue autolysis. Therefore, these pathologies should be excluded in order to consider pre-existing cardiac diseases such as cardiomyopathy based on CTR. Jotterand et al. introduced a new formula for CTR for the diagnosis of cardiomegaly on postmortem CT, taking BMI, age, and sex into account, and suggested that observed dilatation should be considered a subjective parameter when examining PMCT using the proposed formula [93].

CTR, heart weight, and other cardiac measurements are closely linked parameters to evaluate cardiac anatomy, but it remains difficult to relate the heart weight at autopsy to the cardiac measurements assessed with PMI. By analyzing short-axis and four-chamber views with PMMR, Ruder et al. found that a single area measurement reflects actual heart weight measured at autopsy [94]. Similar results were found by Hatch et al. using PMCT and measurement of the left ventricular circumferential area with a linear regression equation [95]. The ventricular mass has also been evaluated using both PMMR and PMCT [96, 97]. Good congruence was found for total heart weight and for both ventricles separately, using PMMR on ex vivo hearts [96] and including the trabecular and papillary musculature. Also, PMCT-based left ventricular shell volume had a good correlation to ventricular mass at autopsy when an adjusted value for myocardial density was used, as shown in the work of Gheorghe et al. [97].

Some studies attempted to measure the ventricle free wall and septum in the same hearts on both PMMR and PMCT to evaluate asymmetrical types of hypertrophy [98–100]. The correlations with autopsy findings were found to be better with PMMR than with PMCT, but overall, imaging methods overestimated the wall thickness, which could lead to an erroneous assumption of cardiac hypertrophy. In addition, measurements on the relative thickness of the ventricular wall at different sites, in order to diagnose segmental types of hypertrophy possibly indicating hypertrophic cardiomyopathy were not recommended [89, 101].

The postmortem radiological diagnosis of other cardiomyopathy-related myocardial abnormalities (i.e., myocardial disarray, fat infiltration). Products related to storage diseases, sarcoidosis, amyloidosis, and idiopathic fibrosis (non-ischemic LV scar) have not been explored systematically yet. The ability to identify these abnormalities depends on the discriminative potential of detecting interstitial changes in the myocardial wall, which is virtually impossible using current postmortem imaging methods. MIA could be helpful in these cases, but clearly it should be further evaluated. Furthermore, components of the conduction system, being a specialized part of the myocardium, cannot be distinguished from the surrounding tissue with the use of PMI and are therefore not targeted by biopsy and require histological examination.

**Fig. 4:**
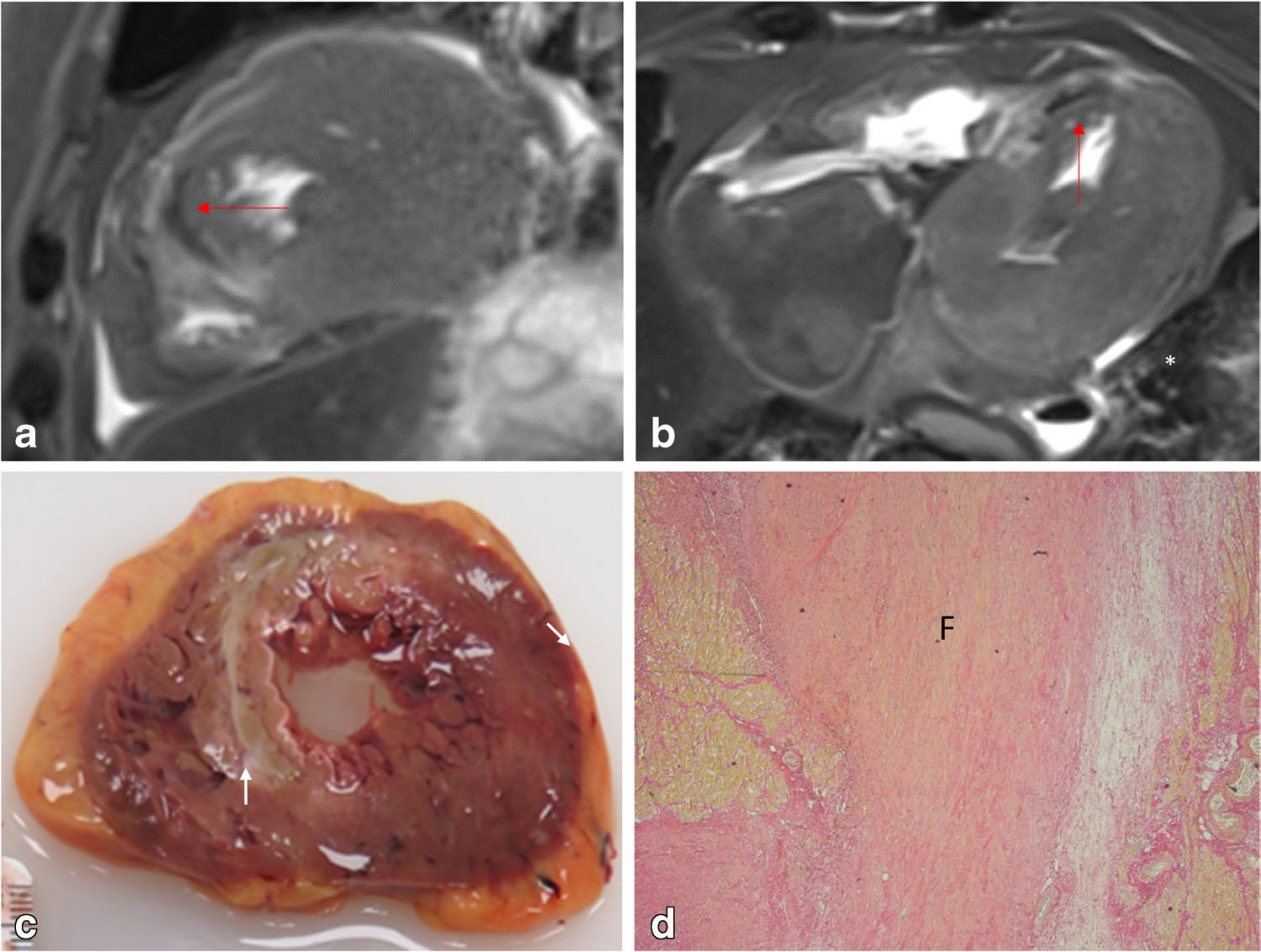
PMMR images of the heart in a case of SCD. Short axial plane T2 (**a**) and longitudinal plane T2 (**b**) showing localized heterogeneity of signal in the ventricular septum with adjacent linear hyposignal (red arrows), suggesting the presence of myocardial injury. **c** Macroscopy and **d** histology of the same area reveal a large area of sclerosis fibrosis (F) of an old myocardial infarction, depicting areas of adjacent vital myocardium (elastic van Gieson stain)

The diagnosis of *chronic ischemic heart disease* could be postulated by the radiological detection of cardiac hypertrophy/dilation and the presence of fibrotic scars (Fig. 4). PMMR could be helpful to detect localized fibrotic scars representing areas of compromised myocardial tissue after the occlusion of arteries [74–76, 80]. Evidently, additional information is provided by the radiological examination of coronary arteries, essentially by PMCTA detecting the signs of CAD. Diffuse (interstitial) fibrosis of the myocardium cannot be visualized since gadolinium as a contrast agent cannot be applied in the postmortem.

### Myocardial tumors

Thus far, PMI has not been applied systematically for the diagnosis of myocardial tumors. For tumors characterized by calcification, PMCT would seem to be suitable [102]. However, the most adaptable PMI modality would be PMMR. For the macroscopic and histopathologic diagnosis of soft tissue tumors, including evaluation of infiltrative growth involving different cardiac structures, an autopsy or a minimally invasive approach with postmortem imaging-guided biopsy is required [103, 104].

*Cardiac cavitary tumors* are rare, and papers on their postmortem imaging are equally scarce. Some single cases have been presented, including a large cardiac fibroma that was clearly seen on PMCT [105]. The most common cardiac tumors, such as myxoma or papillary fibroelastoma, are unlikely to be recognized on PMCT, particularly if small. It should be noted that many of these lesions are usually unable to explain death.

As mentioned in Table 3, small foci of myocarditis or small mural tumors are of limited significance to explain death. At autopsy, such lesions can lead to overinterpretation and misdiagnosis of the cause of death [106], but they likely will not be diagnosed as PMI unless targeted by biopsy. Left ventricular hypertrophy (hypertensive or idiopathic) is difficult to differentiate from (inherited) cardiomyopathy and should also be interpreted in combination with clinical (and family) history.

### Heart valves

The valve diseases involved in SCD that can be diagnosed at autopsy are listed in Table 4.

**Table 4:** Native/prosthetic valve disease and their degree of certainty to explain the onset of SCD (adapted from Basso et al. [2])

Certain	Probable	Uncertain
Mitral valve rupture of papillary muscle or chordae in combination with valvar incompetence and pulmonary edema Thrombotic block or endocarditis vegetation on valve prosthesis Any damage to a valve prosthesis with signs of acute valve incompetence	Calcific aortic valve stenosis with LV hypertrophy and fibrosis Mitral valve prolapse with atrial dilatation or LV myocardial fibrosis	Aortic valve sclerosis without LV hypertrophy Mitral annular calcification Aortic insufficiency (dilated aortic annulus < 4 cm) Mitral valve prolapse without atrial dilatation or LV fibrosis

The components of the heart valves can be seen with PMI without the administration of contrast medium, in the case of the so-called hollow heart chambers (presence of gas enabling virtual evaluation). The use of contrast agents improves visualization [107], and the use of PMCT and PMCTA may prove their value in postsurgical/interventional cases [25, 108–110]. The presence of aortic- or mitral-valve calcifications in clinical and postmortem radiological examination may indicate (stenosing) valvular pathology, but they cannot reliably explain the onset of a SD without additional investigations on the isolated heart [28, 111, 112].

At this stage, there is no literature available to evaluate the value of postmortem imaging of valve diseases in relation to the cause of SD. However, theoretically, it can be anticipated that a thrombotic aortic occlusion superimposed on a prosthetic valve or dislodgments of prosthetic valve components may be targets for PM imaging. Differentiation from postmortem clots remains difficult.

### Pericardial, pleural, and (retro)peritoneal hemorrhage

**Fig. 5:**
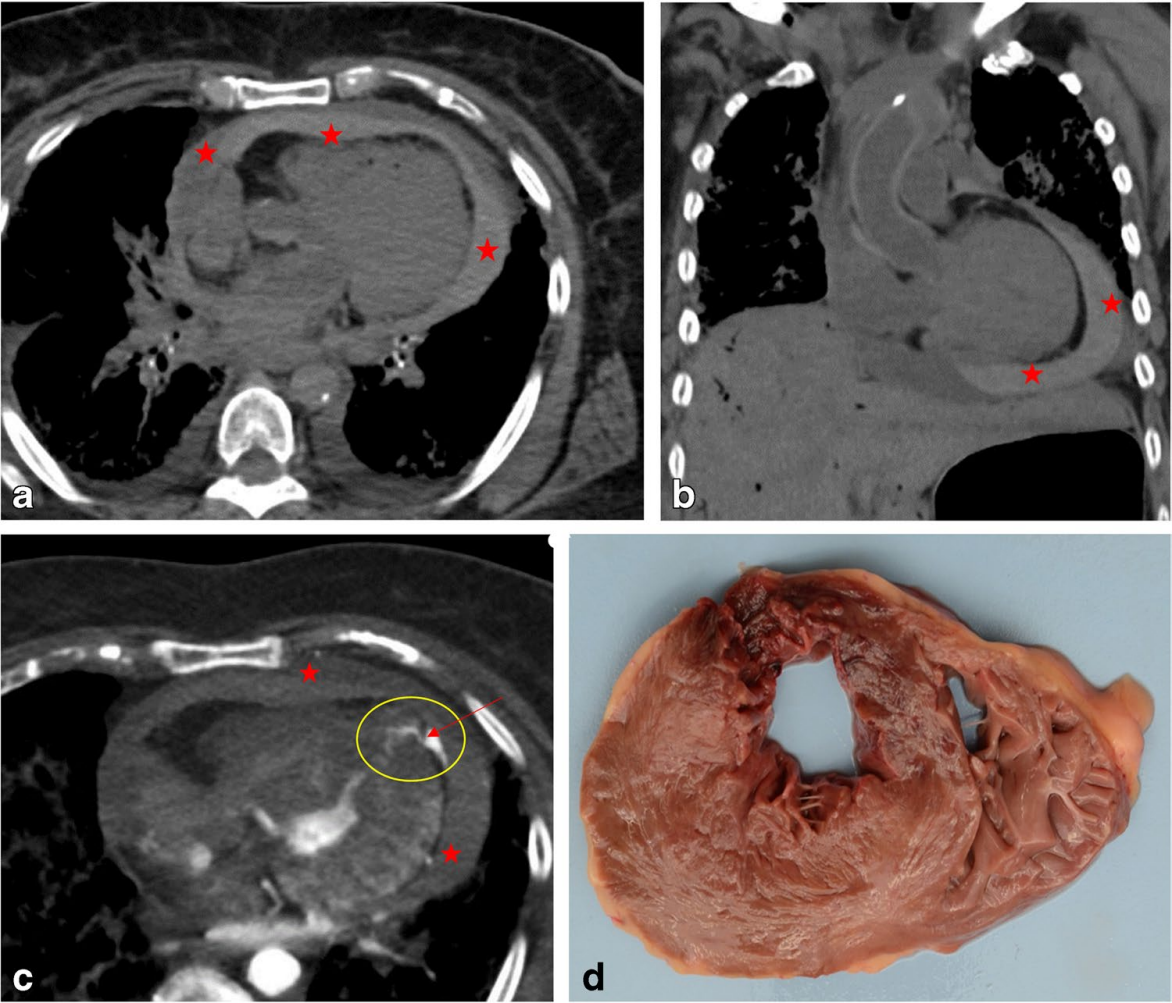
PMCT images of the heart in SCD cases of acute myocardial infarction. Axial (**a**) and coronal (**b**) views showing the presence of hemopericardium (red asterisks). **c** PMCTA, axial plane, also revealing the hemopericardium and additionally a rupture of the apex of the left ventricular wall (yellow ellipse) with a leak of the contrast medium in the pericardium (red arrow). **d** Transversal section of the fresh heart specimen at the level of transmural rupture of the anterior wall in anteroseptal area of hemorrhage due to reperfusion damage of acute myocardial infarction

**Fig. 6:**
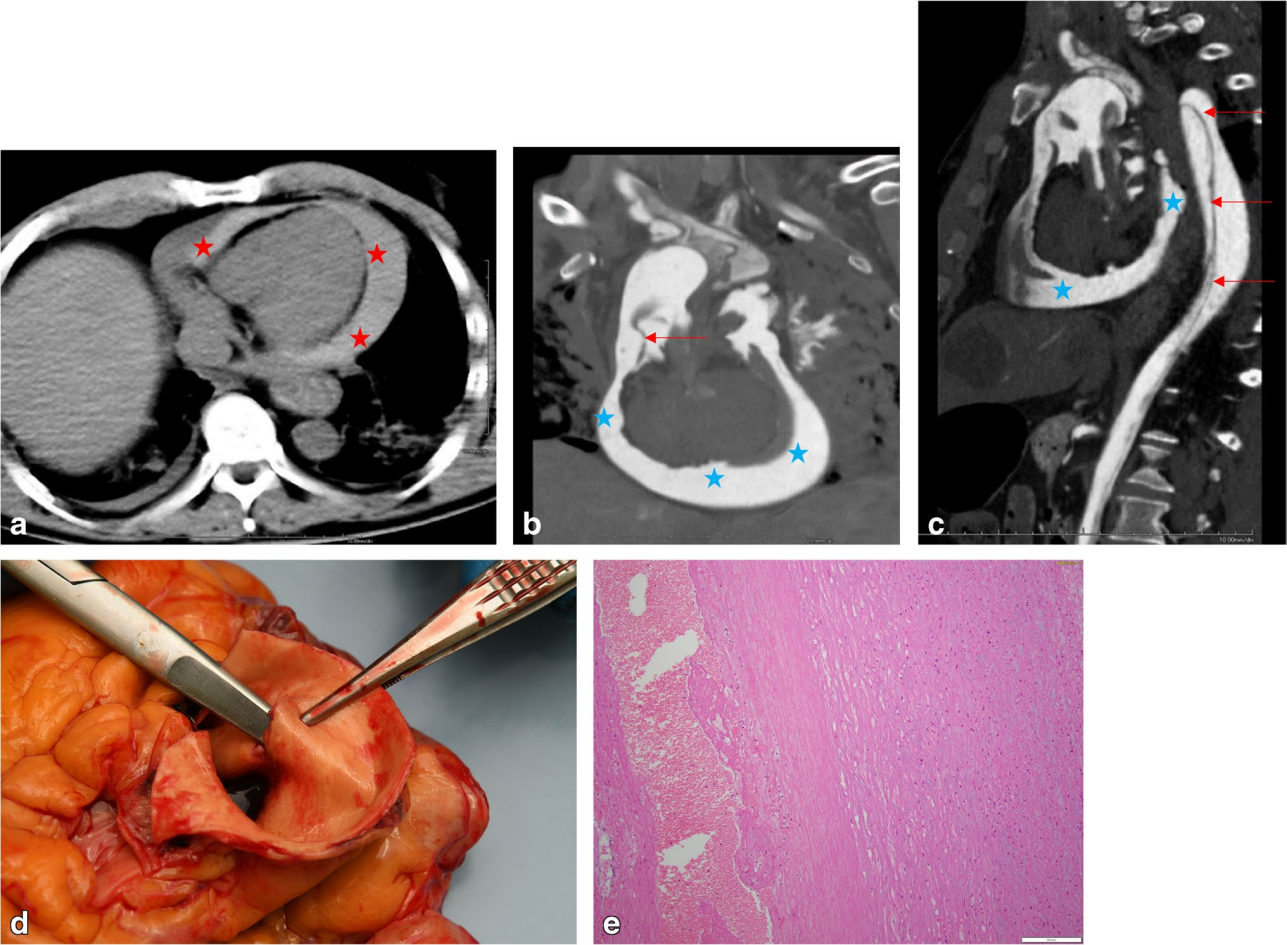
SCD case. **a** Non-contrast PMCT, axial plane, showing hemopericardium (red stars). **b**, **c** PMCTA images, coronal-oblique (**b**) and sagittal-oblique (**c**) planes, with aortic dissection (red arrows) and leak of contrast medium in the pericardium (blue stars) interpreted as caused by the retrograde extension of aortic hematoma. **d**, **e** Macrosopy and histology of the aortic wall showing a dissection channel filled with blood and fibrin and some medial degeneration of the aortic media (HE stain)

Hemopericardium, hemothorax, and hemoperitoneum are well recognized as certain causes of SD [2] (see Table 5) and are readily identifiable on PMI. Hemopericardium at the onset of SD is mostly due to aortic rupture/dissection or to cardiac rupture of infarcted myocardium (Figs. 5 and 6). Hemothorax and hemoperitoneum are mostly related to aortic pathology. In some cases of aortic dissection, the presence of intimal flaps or the so-called double-barrel aorta [113] may suggest the location of entry and exit tears. Without an autopsy, angiography is often required for their visibility [114], even more so since the collapse of the aorta may hamper diagnosis. PMCTA can also help to differentiate between aortic dissection and a ruptured infarct as the cause of a hemopericardium. Shiotani et al. analyzed PMCT appearances of hemopericardium in aortic dissection, named by the author the “hyperdense armored heart” [115], an entity also used for ventricular ruptures complicating myocardial infarction [116]. The armored heart describes a hemopericardium with an inner high-density ring and a lower-density outer ring, producing an armor-like appearance. The proposed mechanism is active pericardial hemorrhage during cardiac motion, during which the blood near the pericardial surface coagulates and blood serum components accumulate at the periphery [115].

**Table 5:** Other causes of SCD (adapted from Basso et al. Virchows Archiv 2017 [2]),

Certain	Probable	Uncertain
Massive pulmonary embolism Hemopericardium (aortic rupture/dissection or cardiac rupture) Cavity tumors obstructing a valve orifice

In the appropriate context, for instance, motor vehicle incidents or penetrating injuries, a traumatic cause of aortic rupture can be assumed. However, determining the etiology of a natural underlying cause of aortic dissection or rupture is usually impossible without a traditional autopsy. In such cases, histological analysis of the aortic wall is needed to identify possible hereditary connective tissue disease [105]. When dissection of the aorta occurs at a young age (< 50 years), with the exclusion of significant trauma, there is always a high suspicion of genetic disease, and sampling of materials for further genetic counseling is recommended. The exclusion of atherosclerotic aortic ulcers and infectious or non-infectious types of aortitis requires a classical autopsy.

Ruptured abdominal aortic aneurysms are easily recognized by PMI by virtue of massive retroperitoneal and sometimes intra-abdominal hemorrhage. Ruptured abdominal aneurysms usually occur at an advanced age (> 60–70) and are nearly always atherosclerotic in nature (atherosclerotic abdominal aneurysm (AAA) or, in rare cases, inflammatory abdominal aneurysms (IAA) [117]). These cases do not require histological verification per se, but histology is advised in atypical cases, such as those involving young individuals. Less common are aortic dissections that propagate from the thoracic aorta to the abdominal segment. The features on imaging are similar to those in the thoracic aorta: a “double-barrel” appearance consisting of a false and true aortic lumen is diagnostic. However, in the absence of angiography, identification hereof may be difficult. Also, dissections of the abdominal aorta are not necessarily fatal and can heal as a fibrotic scar or patent false lumen. A common fatal pathway is when the dissection affects the celiac trunk or mesenteric arteries, thereby causing (fatal) bowel ischemia. These cases, however, are unlikely to present as SCD. Device-related complications (e.g., graft migration, stenosis, thrombosis or infection, and graft endoleaks) require autopsy for a reliable diagnosis, although endoleaks may be visualized by PMCTA [118].

Hemopericardium, hemothorax, and hemoperitoneum should be discriminated at PMI from other fluid collections that relate to pericarditis, malignancy, end-stage renal disease, congestive heart failure, and viral or bacterial infection. A study on the imaging features of postmortem changes in *in-hospital* deaths showed that abdominal fluid occurs more frequently in intensive care unit patients than in other *in-hospital* deaths [119]. The use of Hounsfield units (HU) can help determine the nature of fluid seen on PMCT (Table 6) [120, 121].

**Table 6:** Common Hounsfield units used in CT scan (units based on arbitrarily assigned densities of air and pure water) [120, 121]

Substance	HU
Air	− 1000
Lung	− 500
Fat	− 100 to − 50
Water	0
Muscle	10 to 40
Blood	30 to 80
Soft tissue/contrast	100 to 300
Bone	700 to 3000

Another issue relates to the amount of pericardial fluid that can be considered fatal. Large volumes of fluid can accumulate over a period of time without leading to death, while the rapid accumulation of smaller volumes may lead to cardiac tamponade and death. Determination of the volume of pericardial or pleural fluid on PMI is time consuming. 3D reconstructions or other segmentation techniques can assist [122], but these techniques rely on the ability to differentiate between blood/fluid and soft tissue, which may prove difficult in some cases. Recent studies attempted the use of artificial intelligence (AI) to assist with the determination of the volume of pericardial fluid in CT [123].

Whenever pericardial, pleural, or intraperitoneal blood is noted, the possibility of a CPR-related artifact must be considered. Chest compressions performed often cause rib or sternal fractures, and these may cause hemorrhage in all three compartments. Using PMMR, pleural effusions, intravascular air, and periportal edema have been reported in resuscitated patients [119]. Differentiation between such CPR artifacts and bona fide pathology is often impossible on the basis of PMI alone. Rib fractures related to CPR are usually located anteriorly, and this may help distinguish them from other injuries. Exceptions, however, do occur, and these seem to be more prevalent in the case of the application of mechanic resuscitation devices such as Lucas and AutoPulse [124, 125].

### Pulmonary embolism

Pulmonary embolism is one of the most common causes of SD and is a routine diagnosis in many forensic pathology services. However, it also remains one of the most common conditions that result in a diagnostic discrepancy in studies that compare PMI with conventional autopsy [8]. Pulmonary embolism can be suspected in PMCT by a dilatation of the pulmonary trunk, but this is a subjective sign that is poorly reproducible. It was suggested that PMMR could demonstrate pulmonary thromboembolism in situ [126]. Jackowski et al. suggested that postmortem clots in PMMR show signs of sedimentation due to gravitation, with compartments of lower density containing fibrin and compartments of higher density containing erythrocytes. In contrast, vital clots would appear homogenous, with intermediate signal intensity on T2-weighted images, in direct contact with the vessel wall, and situated within a hypointense layer of sedimented erythrocytes [119]. However, Wagensveld et al. did not find a significant correlation between the postmortem time interval and blood sedimentation or postmortem clotting on PMMR features in *in-hospital* deaths. Clotting was observed more frequently in intensive care unit patients and less in resuscitated patients [119]. Therefore, differentiation from postmortem clots remains a challenging issue. Some secondary (indirect) features, such as lower leg edema, right-sided cardiac dilatation, left ventricle collapse, or the presence of pulmonary infarction, may be helpful in the diagnosis [127, 128]. None of these findings is absolutely diagnostic, and false-positive and false-negative results are common. PMCTA allows the exclusion of PE, but in the case of suspicion, it cannot confirm the presence of PE, and a classical autopsy completed with histological samples is needed.

## Conclusions

Clinical imaging of the heart and great vessels has progressed tremendously, which has had a positive spin-off also for its applications in PMI, especially PMCT and PMMR, and in the first place in forensic medicine. Our review shows that PMI clearly has more limitations to its use in cardiovascular diagnosis than clinical imaging, which enables the use of functional and molecular parameters. Nevertheless, progress has been made in the visualization of cardiac abnormalities that can be related to the (sudden) death of a patient and in the additional use of minimally invasive techniques such as angiography (PMCTA) and image-guided biopsies.

The following conclusions regarding major pathological substrates related to acute cardiovascular death can be drawn from the present literature (see also Table 7):

**Table 7:** Summary of the most important current diagnostic use of postmortem imaging in cases of sudden death related to cardiovascular pathologies

Pathological substrates	Visualisation at postmortem imaging		Comments/pitfalls
	Good^a^	Helpful^b^	
Hemopericardium/hemothorax	PMCT(A) PMMR(A)		• Difficult/impossible to establish the cause of hemorrhage without angiographic methods (MI vs aortic rupture/dissection vs resuscitation artifacts) • Measurement of the volume can be helpful to consider hemorrhage as the cause of death
Aortic rupture/aortic dissection	PMCT(A) PMMR(A)		Histology can be required to elucidate underlying disease (depending on age, circumstance, and family history)
Coronary occlusion		PMCT	Calcifications can be seen but no stenosis or occlusion
		PMCTA	• Acute thrombotic occlusion impossible to be discriminated against by CTO • Opacification and/or degree of stenosis are difficult to evaluate when vessels are highly calcified • The presence of stents in a vessel makes the analysis of the opacification difficult • Diagnostic value increases in combination with findings of acute myocardial injury or “scars” at PMMR
		PMMR	Chemical shift artifacts' lack of sedimentation could indicate coronary thrombus
Coronary stenosis > 75%		PMCTA	• Difficulties to evaluate stenosis in the presence of calcified arteries • Pre-existent stenosis impossible to discriminate from mural thrombus
Congenital coronary lesions	PMCTA PMMRA		Good for the detection of the origin of the coronary artery from the pulmonary trunk, or anomalous origins with the interatrial course of the artery
Acute myocardial infarction		PMMR	• Good detection of early injuries (interstitial edema), better than at autopsy: a topography of edema could be helpful to evaluate • Diagnostic value increases in combination with a coronary occlusion at PMCTA • Pitfall: difficult/impossible to differentiate from other causes of myocardial injuries/postmortem artifacts
Myocarditis		PMMR	Difficult to discriminate from other acute injuries (ischemia, Takotsubo, resuscitations artifacts, and others)
Cardiomyopathies (primary or secondary forms)		PMMR	Histology is needed for the assessment of ventricular wall component makeup in order to classify the type of cardiomyopathy
Idiopathic fibrosis (non-ischemic LV scar)		PMMR	Difficult to discriminate between ischemic and non-ischemic scar
		PMCTA	Stenosis/occlusion of coronaries can be helpful to exclude old infarction
Valvar orifice block due to thrombus, endocarditis’ vegetation on valve prosthesis dislocation		PMCTA	Histology required for differential diagnosis of etiological background
Large cardiac tumors, mural or intracavitary		PMMR PMCTA	• Difficult to discriminate from postmortem clot • Histology required for differential diagnosis
Pulmonary embolism		PMCTA	Difficult to discriminate from postmortem clot
Hemorrhage due to peripheral artery perforation/rupture	PMCTA		Rupture site cannot be demonstrated in all cases

*CTO*, chronic total occlusion; *MPMCTA*, multiphase postmortem CT angiography; *PMCT*, postmortem computer tomography; *PMCTA*, postmortem computer tomography angiography; *PMMR*, postmortem magnetic resonance; *PMMRA*, postmortem magnetic resonance angiography

^a^Certain or highly probable to visualize the cardiovascular pathology (disease)

^b^Helpful to visualize the cardiovascular pathology (disease) but a classical autopsy is needed to confirm the radiological diagnosis

### Coronary artery disease

Ostial and arterial occlusions of large epicardial branches can be effectively diagnosed with the use of PMCTA, but such occlusions cannot be reliably discriminated from old occlusions or artifacts (postmortem clot, etc.). Yet, it must be remembered that any stenosis or obstruction does not mean that the lesion was an acute process, as histology is required for such confirmations. However, a negative PMCTA cannot exclude the coronary cause of death, for example, in the case of a mural but not occluding fresh thrombosis. Therefore, for a certain diagnosis, PMCTA should always be completed by a classical autopsy, including histology of eventual culprit lesions. Histology is also needed to discriminate atherosclerosis-related occlusion from (rare) non-atherosclerotic diseases such as vasculitis. When stenosis of > 75% is found by means of PMCTA, with or without a high coronary calcium score, PMI could provide a probable cause of SCD, but only in cases showing other features of a significant oxygen demand/supply mismatch to the heart. Similar to the autopsy, supportive additional information, such as strenuous exercise or ischemic symptoms, must be retrieved from reviewing the medical history of the patient. Additional PMI findings of the heart, such as signs of pathological hypertrophy and/or dilatation of the heart or the presence of myocardial edema at PMMR, could increase the diagnostic value of the vascular imaging findings. In case of doubt, a histological examination of the heart and a toxicological/biochemical analysis of blood are required.

### Myocardial pathology

PMI enables the visualization of *myocardial edema* with different signal alterations in PMMR, indicating (hyper)acute myocardial injury but without further specification of the underlying myocardial pathology. Topographical distribution of edema (be it subendocardial, subepicardial, transmural, or regional) could give additional information about the cause of the injury, but all of these postmortem artifacts should be considered. MIA techniques followed by histological examination are necessary to determine the pathological nature of findings. Detailed soft tissue characterization, as is required to discriminate the various types of myocardial disease related to SCD, clearly needs further evaluation and standardization before it can be applied reliably and confidently in PMI practice.

Also, the use of a combination of techniques can be helpful: myocardial imaging of acute edema in PMMR, which is associated with occlusion of coronary arteries in PMCTA/PMMRA, can provide a cause of SCD in cases of myocardial infarction.

A diagnosis of acute diffuse myocarditis (any morphological type) is not yet possible with PMI. Further studies are needed to determine the diagnostic role of MIA.

PMI using various methods seems to be helpful in determining heart size and dilatation and therefore, useful for the diagnosis of IHD and some cardiomyopathies. However, the imaging methods overestimate the wall thickness, which might lead to a wrongful assumption of cardiac hypertrophy. Postmortem modifications and agonic period resulting in heart dilatation could be wrongly interpreted as DCM or ACM. At this stage, and in contrast to the novel clinical imaging methods, PMI appears to be still insufficient to visualize differences in components of the ventricular wall and identify fibrosis or fatty infiltrations.

Chronic IHD cannot be diagnosed as the probable cause of SCD by PMCT/PMMR alone but only in combination with angiographic methods and in accordance with the clinical history, external examination, eventual toxicological analyses, etc.

It is impossible to diagnose cardiomyopathies using PMI techniques with a sufficient degree of certitude. The conduction system of the heart cannot be visualized by any of the current postmortem techniques.

### Cardiac valves

Valvular calcifications can be observed, but they cannot explain SCD. Complete acute valvular occlusion due to massive thrombus or vegetation or in the case of prosthetic valve displacement, can be diagnosed by PMI. However, insufficient literature data are available to support the use of PMI in these situations.

### Hemopericardium, hemothorax, and/or hemo(retro)peritoneum

Hemopericardium, hemothorax, and/or hemo(retro)peritoneum in cases of aortic rupture or myocardial infarction, can easily be detected by PMMR(A) and PMCT(A). Artifacts related to the resuscitation should, however, be considered. In hemopericardium, discrimination between ruptured myocardial infarction or aortic rupture/dissection is difficult by PMI without angiographic methods. A detailed examination of etiology (e.g., dating of the lesion in cases of medical responsibility, genetic background in the case of young patients with aortic dissection) requires histological examination and/or genetic analysis.

### Heart tumors

Only calcified amorphous tumors obstructing a valve orifice are likely to be readily seen on PMCT. Distinguishing it from other causes of valvular calcification, such as valvular disease, may, however, be challenging. Theoretically, valvular diseases could be detected by PMCTA/PMMR or PMMRA, but there are no postmortem reports in this field.

### Pulmonary artery embolism

Massive pulmonary embolism can be suspected in PMCTA/PMMR but remains the main source of diagnostic discrepancy when postmortem imaging and conventional autopsy are being compared. A classical autopsy and histology are needed to exclude postmortem clots and determine the age of the thrombus mass.

## Ongoing and future research; prospects for further development

Postmortem imaging of cardiovascular pathologies involved in SD has many limitations. In this review, we identified gaps in the knowledge that need to be filled by new research. The following areas of interest appear to be very promising to increase the diagnostic value of PMI modalities. Several of them, including the evaluation and further development of novel techniques, are currently under investigation.

- Evaluation of the diagnostic value of *myocardial edema* as observed in PMMR in cases of myocardial infarction and to discriminate myocardial infarction from other forms of acute myocardial injury.
- There is a need to establish the radiological characteristics of *vulnerable coronary plaques* in cases of SCD, especially in the younger population. It should be evaluated if the detection of a vulnerable plaque associated with the occlusion of a coronary artery in PMCTA could be considered a vital sign of the occlusion. Postmortem use of new CT technologies (dual-energy CT and photon counting CT) is now under investigation for this purpose. Photon-counting CT, in particular, is considered a promising tool, thanks to its better spatial resolution and improved iodine detection [129, 130]. More information from PMI studies of vulnerable plaque could also further their detection in clinical practice.
- *Pulmonary embolism:* to evaluate the diagnostic value for (positive (PPV) and negative predicted values (NPV)) of secondary features such as lower leg edema, left atrial collapse, and right-sided cardiac dilatation [127]. A diagnostic flowchart that integrates all reported radiological findings [127, 128] and clinical data could increase diagnostic accuracy. The use of image-guided biopsies prior to angiography is likely to be helpful but has not yet been evaluated.
- There is a need to further evaluate the PMI visualization of *specific pathological tissue characteristics of the myocardium* such as fibrosis, fat, and inflammation. At present, PMMR seems to be the most promising radiological tool.
- *MIA* with the use of guided biopsies during PMMR is a promising area receiving intense investigations, and, in addition to myocardial infarction, could be applied to diagnose myocarditis, infiltrative diseases, cardiomyopathies, and aortic pathology. Guided biopsies should be studied also in association with postmortem chemistry and genetic analyses.
- The *artifacts caused by artery calcifications* can hamper the interpretation of coronary artery patency, resulting in an overestimation of atherosclerotic arterial narrowing. Multiple solutions to improve the spatial resolution are available, for example, increasing window width, using iterative reconstruction algorithms or thinner slice thickness, or reducing the field of view. The new generation of CT scanners could provide a different solution, such as modulating the tube potential (kVp) on the Smartscore acquisition.
- *AI and the so-called deep learning techniques(DLT)*: There is little literature on the application of AI and DLT in forensic pathology, especially in the field of cardiovascular pathologies and SD. Up to now, the use of DLT in PMCT was explored only for estimating organ weights and the detection of hemorrhagic pericardial effusion [131, 132].
- Specialized imaging methods like *MR-based diffusion tensor imaging (DTI) or synchrotron radiation-based phase-contrast CT* could potentially solve some of the current diagnostic challenges in myocardial imaging. DTI is a technique sensitive to the anisotropic diffusion of water in tissue, making it possible to evaluate the direction of myocardial fibers and laminar sheets in the myocardium [17]. The direction and dispersion of the myocardial fibers change in structural myocardial disease, e.g., which can impair myocardial function.

The data obtained from DTI could possibly form the basis for developing a 3D Finite element model to test how structural myocardial changes, as can be seen in, e.g., dilation [18], hypertrophy [133], as well as in conjunction with a MI, whether due to fibrosis or possibly acute changes [134, 135], influence myocardial function [133]. For postmortem purposes, however, it is known that prolonged postmortem intervals could influence the DTI results [17, 134], and due to the experimental nature, the evaluation of myocardial architecture is still a matter of debate [136]. Synchrotron radiation-based phase-contrast CT, an experimental CT-based technique, has proven useful for the visualization of the conduction system [137] and can possibly be an aid when evaluating conduction system-related disease. These methods are mainly used experimentally, and further research is needed in order to determine their usefulness, e.g., in a postmortem setting.

## Abbreviations

AECVP: The Association for European Cardiovascular Pathology
AUC: Area under the curve
AI: Artificial intelligence
ACAD: Acute coronary artery disease
ACM: Arrhythmogenic cardiomyopathy
AV: Atrioventricular
CACS: Coronary artery calcium scoring
CAT: Calcified amorphic tumor
CI: Confidence interval
COD: Cause of death
CDI: Cardiac dilatation index
CPR: Cardiopulmonary resuscitation
CTR: Cardiothoracic ratio
CTO: Chronic total occlusion
DCM: Dilated cardiomyopathy
DLT: Deep learning techniques
DTI: Diffusion tensor imaging
HU: Hounsfield units
IHD: Ischemic heart disease
LBB: Left bundle branch
LCA: Left coronary artery
LV: Left ventricle
MI: Myocardial infarction
MIA: Minimally invasive autopsy
MPMCTA: Multiphase postmortem CT angiography
PE: Pulmonary embolism
PMCT: Postmortem computer tomography
PMCTA: Postmortem computer tomography angiography
PMMR: Postmortem magnetic resonance
PMMRA: Postmortem magnetic resonance angiography
RBB: Right bundle branch
SD: Sudden death
SCD: Sudden cardiac death
VF: Ventricular fibrillation
